# Evaluation of hospital staff’s perceived quality of librarian-mediated literature searching services*,†

**DOI:** 10.5195/jmla.2017.201

**Published:** 2017-04

**Authors:** Sandra McKeown, Shauna-Lee Konrad, Jill McTavish, Erin Boyce

## Abstract

**Objective:**

The research evaluated the perceived quality of librarian-mediated literature searching services at one of Canada’s largest acute care teaching hospitals for the purpose of continuous quality improvement and investigation of relationships between variables that can impact user satisfaction.

**Methods:**

An online survey was constructed using evidence-based methodologies. A systematic sample of staff and physicians requesting literature searches at London Health Sciences Centre were invited to participate in the study over a one-year period. Data analyses included descriptive statistics of closed-ended questions and coding of open-ended questions.

**Results:**

A range of staff including clinicians, researchers, educators, leaders, and analysts submitted a total of 137 surveys, representing a response rate of 71%. Staff requested literature searches for the following “primary” purposes: research or publication (34%), teaching or training (20%), informing a policy or standard practice (16%), patient care (15%), and “other” purposes (15%). While the majority of staff (76%) submitted search requests using methods of written communication, including email and search request forms, staff using methods of verbal communication, including face-to-face and telephone conversations, were significantly more likely to be extremely satisfied with the librarian’s interpretation of the search request (*p*=0.004) and to rate the perceived quality of the search results as excellent (*p*=0.005). In most cases, librarians followed up with staff to clarify the details of their search requests (72%), and these staff were significantly more likely to be extremely satisfied with the librarian’s interpretation of the search request (*p*=0.002).

**Conclusions:**

Our results demonstrate the limitations of written communication in the context of librarian-mediated literature searching and suggest a multifaceted approach to quality improvement efforts.

## INTRODUCTION

Librarian-mediated literature searching remains one of the key services that health sciences librarians provide [1, 2]. The evidence suggests that library services—in which literature searching is prominent—save clinicians time [3–7], influence clinical decision making [3, 5–8], and positively impact patient outcomes [5, 7, 9, 10]. Despite this demonstrated value, many libraries struggle to maintain their position in parent organizations due to budgetary constraints and the common misperception that the Internet and related evolving technology obviate the need for librarians [11, 12]. In this difficult climate, it is imperative that health sciences libraries continually strive to improve their methods.

Library research shows that efforts to improve the quality of the librarian-mediated literature search have been ongoing, and a number of studies offer valuable insight into patron preferences and potential areas for improvement. Previous surveys have revealed that most participants prefer search request forms that solicit an expected number of citations [13] and results that are delivered by a handheld device or web interface [3]. Other surveys have found that the timeliness of delivering search results is important to patrons [14] and that decreasing the number of returned citations while increasing relevance is an important area for improvement [13, 15]. A recent study evaluating literature search services by auditing service metrics at a large health library system concluded that improvement is necessary in the areas of data collection by librarians (e.g., time spent and resources searched), service promotion, administration of user-satisfaction surveys, and overall service standards [1]. Recommended improvements to the literature search service include facilitation of smooth and instant access to full text, greater levels of communication to improve search precision, and increased promotion of the service [6, 13, 15].

Researchers evaluating hospital library literature searching services have concluded that continuous user satisfaction surveys are necessary for ongoing quality improvement [15]. The goals of this research project were to ascertain the perceived quality of the literature searching service that librarians offered at a large acute-care teaching hospital and to investigate relationships between variables that can impact user satisfaction. Evaluations of literature search services to date have not statistically analyzed whether levels of satisfaction depend on variables such as professional designation or methods of communication. The authors report on a range of factors cited as important in research about literature searching services, including perceived relevance of results and satisfaction with the formatting, delivery, and timeliness of results. This research demonstrates that clear areas for improvement can be found even when patrons are highly satisfied with the service and that methods of communication and follow-up clarification with the librarian are related to user satisfaction.

## METHODS

### Study participants

The London Health Sciences Centre (LHSC) is one of Canada’s largest acute-care teaching hospitals, with nearly 15,000 physicians and staff serving more than 1 million patients annually. At the time of this evaluation, LHSC’s health sciences library employed 7 clinical librarians, 2 library technicians, and a library manager, with a mandate of providing information services to physicians and staff to support the hospital’s patient care, education, and research activities.

With over 600 librarian-mediated literature searches conducted per year in 2014 and 2015, literature searching represents LHSC library’s most popular professional service. LHSC staff and physicians (hereafter referred to as staff) request searches for the purposes of patient care, administrative decision making, research, and educational purposes. For each literature search requested, a clinical librarian executes a topical search in any number of relevant resources. Literature search results can include references to journal articles, practice guidelines, books, gray literature, and websites.

### Study design

The survey study employed the critical incident technique, which has been shown to be effective in evaluating the impact of library services [16]. The critical incident technique encourages participants to recall descriptions of actual events rather than to speculate about abstract events or situations [17].

In the current study, a critical incident was defined as any literature search requested by staff and conducted by a librarian from the LHSC. Participants were asked to base their survey responses on the results of one particular literature search service experience. Systematic review searches and literature searches conducted for the purpose of current awareness initiatives were excluded.

### Sample size and recruitment

In the critical incident technique, sample size calculations are based on the number of critical incidents. Using the previous year’s annual literature search statistics, it was projected that LHSC’s clinical librarians would conduct approximately 620 searches during the study period. Using the sample size calculator recommended by Weightman et al. [18], the researchers determined that a sample size of 237 (approximately one-third of the entire population) would be required for 95% confidence. The sample size was confirmed by consulting a quick reference table for determining sample size for research activities [19]. The study utilized a systematic sampling technique [20], where 1 out of 3 recipients of literature search results were sent an invitation to participate in the survey.

Literature search results sent to staff between February 1, 2014, and January 31, 2015, were eligible for evaluation. Staff used the usual methods (phone, face-to-face, email, or literature search request form) to submit literature search requests to librarians. Upon completing the search and sending the results to staff, librarians entered the details of the search request into the library’s statistics database. Using this database, the study administrator emailed every third recipient of literature search services an invitation to participate in the study, including the survey link, the letter of information, and their unique identifying number. Consent to participate was assumed upon voluntary completion of the study survey. Librarians conducting the literature searches were blinded as to which of their patrons were included in the study, since the first invitation to a search recipient was based on a randomly generated number that was only known by the study administrator. The study was approved by the Research Ethics Board at Western University.

Following Weightman et al.’s recommendations for best practices for library impact research, several steps were taken to increase response rate, including advance notification, an incentive, and reminders [18]. Study participants who completed the survey were compensated with a $5 LHSC meal card. Upon submission of the survey, participants were directed to a separate online form, where they entered their unique identifying number to receive their compensation. In this way, survey responses were kept completely separate from participants’ identifying information. Two reminder emails were sent at 7-day intervals to staff who had not yet sought their compensation. Funding for the study incentives was generously provided by the Canadian Health Libraries Association/Association des bibliothèques de la santé du Canada Chapter Initiatives Fund.

### Survey tool

The researchers developed an online survey tool using SurveyMonkey (supplemental appendix). Using a combination of closed- and open-ended questions, the short survey solicited details about the submission of the request, interactions with the librarian, perceived quality of the search results, and overall impressions of the provided service. Participants were also asked to provide suggestions for how the results and service could have been improved. Standard survey design strategies were utilized, and steps were taken to increase survey validity [21].

The tool underwent a multipronged revision process, including review by survey design experts, library colleagues, and representatives of the study population. A 1-month pilot study was conducted to reveal weaknesses in the survey tool or the study process. Problems with the survey or the study process identified during the multipronged review process or the pilot study were corrected prior to the initiation of the study [21]. Following the survey revision, the calculated Cronbach’s alpha showed a reliability of 0.80, consistent with “good reliability.”

### Data analysis

Individual survey results were only viewable by the study administrator, who collated the results and removed identifying data such as job title, department, or mention of a specific librarian.

Descriptive statistics were used to evaluate results from closed-ended questions, and differences between variables were analyzed for statistical significance using chi square tests. Two different coders analyzed responses to open-ended questions. Each coder developed a coding scheme comprising key themes. The coders then met to compare schemes, and differences were resolved via a consensus process. For analysis purposes, the coders agreed to combine the answers to the questions pertaining to suggestions for improving the search results and service due to the amount of overlap in the answers.

## RESULTS

### Participant demographics

Staff submitted a total of 137 surveys over the 1-year study period, representing a response rate of 71%. By professional designation, physicians (including residents and fellows) represented the largest group to respond (35%), followed by nurses (27%), allied health professionals (23%), and professional designations in the “other” category (15%). Respondents who selected the “other” category for professional designation most often self-identified as scientists, researchers, or research coordinators.

When staff were asked approximately how many literature searches they had requested from the library in the past 12 months (including the search request they were being asked to evaluate), 19% of staff indicated that this was their first search request in the past year. Sixty-one percent of staff indicated that they had requested between 2 and 6 searches, and 12% between 8 and 15 searches. Four percent (each) of staff had requested 20 and 30 literature searches in the past year. The average number of searches requested in the past 12 months (including the search requests they received the survey about) was 5.

### Purpose of the search request

**Figure 1:**
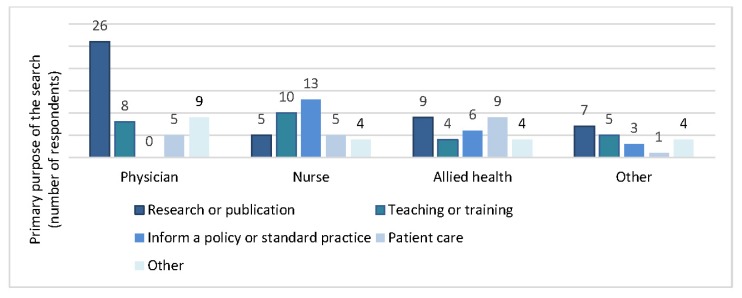
Primary purpose of the search request by professional designation

Staff requested literature searches for a variety of “primary” purposes, with “research or publication” being the most commonly reported reason (34%), followed by “teaching or training” (20%) and “inform a policy or standard practice” (16%). Interestingly, patient care (including patient care in general or the clinical care of a particular patient) was the least likely reason for requesting a search (15%). Respondents who requested searches for “other” purposes (15%) specified different reasons for their search requests, including master’s or school work, quality improvement projects, media campaigns, and personal knowledge. The primary purpose for requesting a literature search varied across physicians, nurses, allied health professionals, and others (Figure 1). Sixty-eight percent of respondents tried to find information themselves before requesting a search from the library.

### Most important aspect of the search request

**Figure 2:**
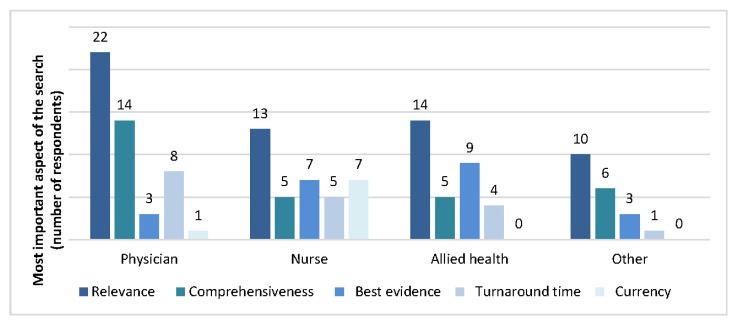
Most important aspect of the search request by professional designation

When asked to prioritize the most important aspect of their search request, most staff specified that the “relevance of search results” was most important (43%), while few staff indicated that the “currency of search results” was most important (6%). The most important aspect of the search request varied among physicians, nurses, allied health professionals, and others (Figure 2). Allied health professionals were more likely than any other group to rate “best level of evidence” as the most important aspect of the search and were significantly more likely than physicians to do so (*p*=0.007).

### Method of submitting the initial search request

**Figure 3:**
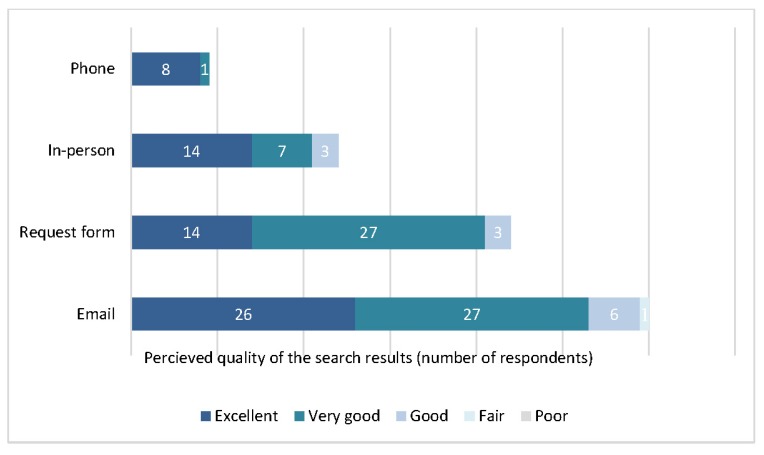
Perceived quality of the search results by method of submitting the search request

The most popular method for submitting a literature search request was via email (44%), followed by using print or online literature search request forms (32%). Requests were submitted less often by phone (18%) and in-person (7%). However, staff were significantly more likely to be “extremely satisfied” with the librarian’s interpretation of the search request (*p*=0.004) when the initial request was submitted verbally, by speaking with a librarian in-person or over the phone, in comparison to using email or a search request form. Staff were also significantly more likely to rate the perceived quality of the search results as “excellent” (*p*=0.005) when the initial request was submitted using methods of verbal communication in comparison to written communication (Figure 3).

### Follow-up clarification

After the initial search request was submitted, follow-up communication occurred with the majority of search requests (72%) in order for the librarian to clarify the search parameters. Follow-up clarification was most likely to occur when the search request was submitted by phone (100%, 9/9), followed by email (83%, 50/60) and in-person (71%, 17/24). Follow up was least likely to occur when the search request was submitted using a search request form (52%, 23/44). Staff were significantly more likely to be “extremely satisfied” with the librarian’s interpretation of the search (*p*=0.002) when the librarian followed up (either verbally or by email) to clarify the initial search request. However, staff were not significantly more likely to rate the perceived quality of the search results as “excellent” when the librarian followed up for clarification.

### Relevance and usefulness of the search results

When staff were asked how satisfied they were with the librarian’s interpretation of the search request, 54% indicated “extremely satisfied,” 42% “very satisfied,” 3% “moderately satisfied,” and 1% “slightly satisfied,” with no staff responding “not at all satisfied.” When staff were asked what percentage of the search results were relevant to their topics, 45% of staff indicated 75%–100%, 34% indicated 50%–74%, and 22% indicated that less than 50% of the search results were relevant. Staff who received 75%–100% relevant results were significantly more likely (*p*=0.003) to be “extremely satisfied” with the librarian’s interpretation of the search request than staff receiving less than 75% relevant results. Staff who received 75%–100% relevant results were also significantly more likely to rate the perceived quality of the search results (*p*=0.00002) and the search service (*p*=0.0009) as “excellent.” Despite the occurrence of irrelevant results, 92% of staff felt that the number of search results that they received was “just right,” while 3% felt they received “too few” and 5% “too many.”

**Figure 4:**
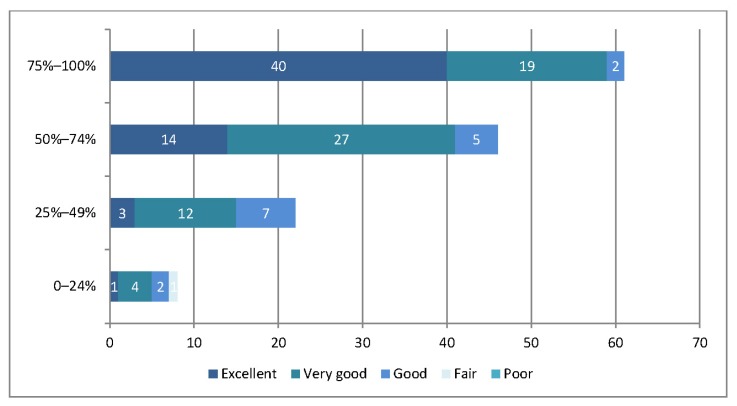
Usefulness of the search results by percentage of relevant search results

When staff were asked to rate the usefulness of the search results that they received, 42% rated the usefulness of the results as “excellent,” 45% as “very good,” 12% as “good,” and 1% as “poor,” with no staff responding “fair.” The usefulness of the search results was positively associated with the percentage of relevant results received (Figure 4). Fifty-six percent (27/48) of physicians rated the usefulness of the search results as “excellent,” compared to 44% (14/32) of allied health professionals and 30% of both nurses (11/37) and others (6/20). Twelve percent of staff indicated that they were aware of additional literature that they expected to see in the search results, accordingly: 25% (5/20) of others, 19% (9/48) of physicians, 6% (2/32) of allied health professionals, and 3% of nurses (1/37).

### Format and delivery of search results

Our literature search results are often provided to staff in a Word document as an alphabetized reference list, including abstracts. When asked if they were satisfied with the provided layout or format of the literature search results, 93% of staff indicated “yes,” 1% indicated “no,” and 5% indicated “unsure.” Staff responding “no” or “unsure” were asked to explain. One staff member commented: “there did not seem to be any systematic organization. For me it would be best if results were sent in a spreadsheet/reference manager format.”

Instructions on obtaining the full text of results are provided at the discretion of individual librarians and can include a link to the library’s online article request form or ask staff to indicate which references they would like in full text. When asked if they were satisfied with the instructions provided for obtaining the full text of search results, 85% of staff indicated “yes,” 2% indicated “no,” 9% indicated “unsure,” and 4% did not respond. When asked to explain, staff who responded “no” commented that direct links to the full-text search results would be preferred: “a direct link would be helpful. I’m still not sure how to get full-texts. A physician kindly helped me”; “would be helpful to have links attached if possible to the articles themselves.”

### Perceived quality of the search results and service

**Figure 5:**
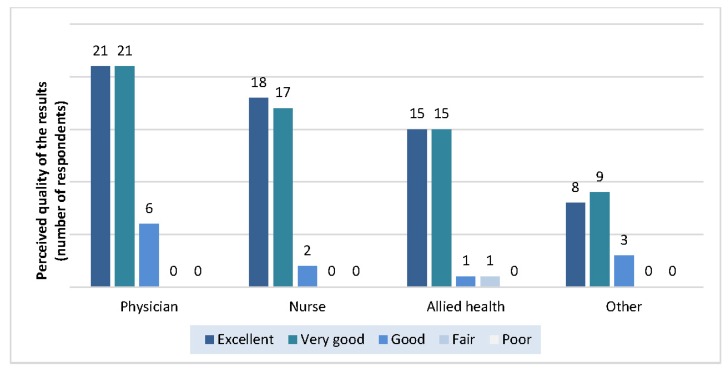
Perceived quality of the search results by professional designation

The perceived quality of the search results was similar among physicians, nurses, allied health professionals, and others (Figure 5). Interestingly, staff were significantly more likely (*p*=0.002) to rate the perceived quality of the search service as “excellent” (64%) than they were to rate the perceived quality of the results as “excellent” (45%).

Staff responses to open-ended questions about how to improve the quality of the search results and service revealed several possible areas for improvement (Table 1). The top three themes for improvement all related to communication between librarians and staff.

**Table 1:**
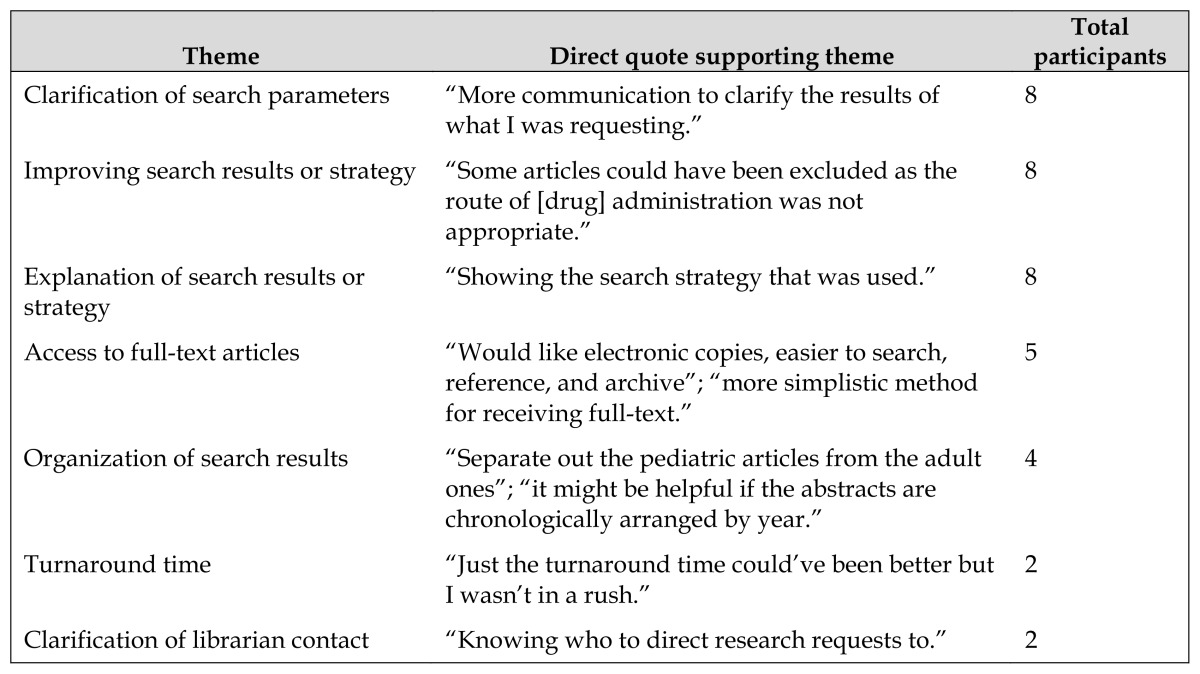
Suggestions for improving the search results and service

Theme	Direct quote supporting theme	Total participants
Clarification of search parameters	“More communication to clarify the results of what I was requesting.”	8
Improving search results or strategy	“Some articles could have been excluded as the route of [drug] administration was not appropriate.”	8
Explanation of search results or strategy	“Showing the search strategy that was used.”	8
Access to full-text articles	“Would like electronic copies, easier to search, reference, and archive”; “more simplistic method for receiving full-text.”	5
Organization of search results	“Separate out the pediatric articles from the adult ones”; “it might be helpful if the abstracts are chronologically arranged by year.”	4
Turnaround time	“Just the turnaround time could’ve been better but I wasn’t in a rush.”	2
Clarification of librarian contact	“Knowing who to direct research requests to.”	2

Eighty-five percent of staff were “extremely likely” to recommend the literature search service to others, 14% were “likely,” and 1% were “neutral.” None of the respondents were “unlikely” or “extremely unlikely” to recommend the service to others.

## DISCUSSION

### Hospital activities supported by search services

Despite the growing amount of evidence that demonstrates the value and impact that literature searching services contribute to patient care activities, hospital staff at LHSC were least likely to request literature searches for patient care purposes. One possible explanation for this finding is that staff might find it easier to obtain patient care information in comparison to other types of information. For example, throughout the duration of this study, LHSC staff had access to point-of-care tools such as UpToDate and drug resources including RxTx, Micromedex, and Lexicomp. Staff might be able to bypass using more complicated bibliographic databases such as PubMed and CINAHL when conducting a search for patient care by utilizing point-of-care tools and drug resources that are often quicker and easier to use.

Bibliographic databases, however, are crucial resources when conducting literature searches for research and publication purposes. It also seems plausible that staff might be more likely to request a librarian-mediated literature search when comprehensive searching is a necessity. This could explain why the greatest number of respondents in this study requested a literature search for research and publication purposes.

Finally, organizational activities and priorities during the one-year study period might have influenced the types of literature searches that staff requested. It is worth noting that LHSC implemented transformative work in 2014 to advance toward an electronic patient record and evidence-based standardization of care [22]. Key elements of this work, including moving to computerized provider order entry, impacted all clinical services and disciplines.

### Verbal versus written methods of communication

The results demonstrates that hospital staff are significantly more satisfied with aspects of the literature searching service when searches are requested either by speaking with a librarian in person or over the phone in comparison to using email or request forms. It is unclear from these findings whether this relationship can be attributed to the quality of information that is exchanged using verbal versus written methods of communication. After all, it is possible that there is a difference between the type of patrons who prefer a more human touch when contacting the library and the type of patrons who prefer less personal methods of communication. The difference between these two groups might be intricately related to user satisfaction, where patrons who preferred to see or speak with a librarian might inherently place a higher value on the service.

To provide more insight into these findings, future research could examine the quality of information that is exchanged when patrons use verbal versus written or electronic methods of communication to request literature searches. For example, are patrons more likely to provide detailed information about their search requests when they speak with a librarian verbally and do not have to type everything out in an email or online request form? Are librarians more likely to confirm aspects of the literature search request when they speak to a patron in comparison to using email? Future research could investigate these questions to help librarians understand how methods of communication impact the success of the literature search service.

### Limitations of written and electronic methods of communication

When using written and electronic methods of communication during literature search transactions, a previous study found that a systematic approach to conducting the email reference interview was most successful, leading the author to develop an electronic request form to make the process more efficient [23]. The details collected in this electronic request form are similar in content to the literature search request form that LHSC library uses, which elicits details from staff about their topics including the purpose of the search request; any known articles on the topic; the preferred age groups, publication types, and number of results (in predefined ranges); and the time period to be searched. Interestingly, this study found that staff using the literature search request forms were actually least satisfied with the librarian’s interpretation of the search and the perceived quality of the search results. These findings might be related to the fact that follow-up clarification was least likely to occur when staff submitted their requests using a literature search request form, given that follow up was found to significantly increase satisfaction with the librarian’s interpretation of the search request (but not the perceived quality of the search results). Another explanation might be that literature search request forms are inherently limited in their ability to effectively accommodate a wide range of information needs (background to foreground questions, clinical to nonclinical topics) that are requested for various purposes or activities (research, teaching, policy development, patient care, etc.). This seemed to be the case in a multicenter study that found a minimally structured form was favored in comparison to an evidence-based medicine structured form to facilitate literature search requests from clinicians, researchers, and health sciences students [24].

### Improvement of user satisfaction

While the findings of this study indicate that hospital staff are highly satisfied with the quality of the literature search results and service that librarians at LHSC provide, opportunities for improvement were identified, particularly in the area of communication between librarians and staff. It is recommended that librarians follow up with patrons to clarify their search requests, as this was found to improve levels of satisfaction with the librarian’s interpretation of the request. Given that staff were more satisfied with the perceived quality of the search results when they submitted their requests to a librarian in person or over the phone, it may be worthwhile to follow up verbally with patrons when clarifying search requests, if possible. Communication can also be improved at the point of delivering search results by explaining the search strategy used and providing clear and concise instructions about how to obtain full-text content. This research demonstrates the intricate relationships between variables of communication that can impact librarian processes and user satisfaction, highlighting the need for a multifaceted approach to quality improvement efforts.

### Limitations

Results of this survey may be generalizable to other health sciences libraries providing literature search services to similar patrons. Hospital librarians are encouraged to use our survey instrument (supplemental appendix) to assess the perceived quality of their own literature searching service. One limitation of this study is that the findings of this survey do not directly assess the impact of the literature searching service on patient care, which is an important measure for hospital libraries. Future surveys could combine questions from the supplemental appendix and the survey developed by Farrell and Mason [10] to fulfill this need.

Another limitation of this study is the potential for participant recall bias when completing the survey about their search requests. This limitation was somewhat mitigated in our study because participants could (if they chose to) reference their search results as they completed the evaluation survey.

Voluntary surveys are susceptible to response bias, where respondents with particularly strong feelings, whether positive or negative, may be more inclined to respond. However, measures were taken to increase the likelihood that staff would participate in the survey, including advance notification of the survey, offer of a $5 LHSC meal card to staff as an incentive to complete the survey, and 2 follow-up reminder emails to staff who were invited to participate in the survey.

This study does not compare characteristics of responders and nonresponders such as professional designation, purpose of the search, or method of submitting the request. Although a 70% response rate is considered to be “very good” for generalizability [25], comparing characteristics of responders and nonresponders could have revealed whether significant differences exist between the 2 groups that might impact the validity of the survey findings.

## Supplemental File

Appendix Literature search evaluation survey
