# Supplementary material for: Evaluation of hospital staff’s perceived quality of librarian-mediated literature searching services
Source: J Med Libr Assoc. 2017 Apr;105(2):120–31. doi: 10.5195/jmla.2017.201 (PMC5370602; doi:10.5195/jmla.2017.201)
Supplement: Appendix [file jmla_apr17_mckeown_app.pdf]

JMLA Journal of the Medical Library Association

SC

Supplemental content to
J Med Libr Assoc. Apr;105(2): dx.doi.org/10.5195/jmla.2017.201
www.mlanet.org
© McKeown, Konrad, McTavish, Boyce 2017

Evaluation of hospital staff's perceived quality of librarian-mediated literature searching services

Sandra McKeown, MLIS; Shauna-Lee Konrad, MLIS; Jill McTavish, PhD, MLIS; Erin Boyce, MLIS

APPENDIX

Literature search evaluation survey

The purpose of this survey is to gauge the perceived quality of the search results and literature search service provided by clinical librarians at the London Health Sciences Centre (LHSC).

The survey will take less than 5 minutes to complete, and you will be compensated with a $5.00 LHSC meal card, generously funded by a grant from the Canadian Health Libraries Association/Association des bibliothèques de la santé du Canada.

When answering, please consider **only the literature search referenced in the email** that provided you the survey link.

Your thoughtful participation is greatly appreciated and will contribute to the improvement of the literature searching service at the LHSC Health Sciences Library. Your responses will be kept completely anonymous and will **not** be attached to personal identifiers.

Questions or concerns about the survey may be directed to: LitSearchEvaluationStudy@lhsc.on.ca.

1. What best describes your professional designation? (Check only one.)
O Allied health professional
O Nurse
O Physician
O Not applicable
O Other (please specify)

---

2. What best describes your role/position at LHSC? (Check all that apply.)
O Clinical staff
O Leadership (coordinator, manager, etc.)
O Program coordinator/educator/facilitator
O Consultant/analyst
O Researcher
O Resident/Fellow
O Technologist
O Scientist
O Student
O Other (please specify)

---

3. Approximately how many searches have you requested from the library in the past 12 months, including this search request? (Please provide a numeric value.)

JMLA Journal of the Medical Library Association

SC
Supplemental content to
J Med Libr Assoc. Apr;105(2): dx.doi.org/10.5195/jmla.2017.201
www.mlanet.org
© McKeown, Konrad, McTavish, Boyce 2017

4. Did you search for literature yourself before requesting this search?
O Unsure
O Yes
O No

5. What was the *primary* purpose for requesting this search? (Check only one.)
O Patient education
O Clinical care of a particular patient
O Patient care in general
O Inform a policy or standard practice
O Research or publication
O For teaching or training
O Other (please specify)

---

6. How did you *initially* provide the details of this search request to the librarian?
O Email
O Literature search request form (online or print)
O Voice mail
O Telephone conversation
O Face-to-face conversation

7. After the initial request, did any follow-up communication occur with the librarian to clarify details of this search request?
O Unsure
O Yes
O No

8. How satisfied are you with the librarian's interpretation of this search request?
O Not at all satisfied
O Slightly satisfied
O Moderately satisfied
O Very satisfied
O Extremely satisfied

9. How do you feel about the number of search results received?
O Too many
O Too few
O Just right

10. Approximately what percentage of the search results was relevant to your topic?
O 0–24%
O 25%–49%
O 50%–74%
O 75%–100%

SC
Supplemental content to
J Med Libr Assoc. Apr;105(2): dx.doi.org/10.5195/jmla.2017.201
www.mlanet.org
© McKeown, Konrad, McTavish, Boyce 2017

11. Are you aware of additional literature that you expected to see in the search results, but did not (e.g., articles, guidelines, books)?
O Unsure
O Yes
O No

12. Were you satisfied with the layout/format of your search results?
O Unsure
O Yes
O No (Please explain.)

---

13. Were you satisfied with the instructions on how to obtain the full text of search results?
O Unsure
O Yes
O No (Please explain.)

---

14. What was the most important aspect of this search request? (Check only one.)
O Relevance of search results
O Currency of search results
O Receiving the best level of evidence available
O Comprehensiveness of search results
O Turnaround time of search results
O Other (Please specify.)

---

15. What other aspects of this search request were important? (Check all that apply.)
O Relevance of search results
O Currency of search results
O Receiving the best level of evidence available
O Comprehensiveness of search results
O Turnaround time of search results
O Not applicable
O Other (Please specify.)

Quality of search results: These 5 questions pertain to the literature search results.

16. How would you rate the usefulness of the search results you received?

Poor	Fair	Good	Very good	Excellent
O	O	O	O	O

17. How satisfied are you with the range of publication dates in the search results you received?

Not at all satisfied	Slightly satisfied	Moderately satisfied	Very satisfied	Extremely satisfied
O	O	O	O	O

JMLA Journal of the Medical Library Association

SC
Supplemental content to
J Med Libr Assoc. Apr;105(2): dx.doi.org/10.5195/jmla.2017.201
www.mlanet.org
© McKeown, Konrad, McTavish, Boyce 2017

18. How satisfied are you with the publication types included in the search results you received (e.g., reviews, guidelines, clinical trials, books)?

Not at all satisfied	Slightly satisfied	Moderately satisfied	Very satisfied	Extremely satisfied
O	O	O	O	O

19. Overall, how would you rate the quality of the search results you received?

Poor	Fair	Good	Very good	Excellent
O	O	O	O	O

20. Please provide suggestions for how your search results could have been improved.

Quality of service: These 6 questions pertain to the literature search service.

21. How satisfied were you with the turnaround time of the search results?

Not at all satisfied	Slightly satisfied	Moderately satisfied	Very satisfied	Extremely satisfied
O	O	O	O	O

22. How satisfied were you with the comprehensiveness of the resources searched by the librarian (e.g., databases searched)?

Not at all satisfied	Slightly satisfied	Moderately satisfied	Very satisfied	Extremely satisfied
O	O	O	O	O

23. How satisfied were you with the librarian's explanation of the search results?

Not at all satisfied	Slightly satisfied	Moderately satisfied	Very satisfied	Extremely satisfied
O	O	O	O	O

24. How satisfied were you with your interactions with the librarian?

Not at all satisfied	Slightly satisfied	Moderately satisfied	Very satisfied	Extremely satisfied
O	O	O	O	O

25. How likely are you to recommend the literature search service to others?

Extremely unlikely	Unlikely	Neutral	Likely	Extremely likely
O	O	O	O	O

26. Overall, how would you rate the quality of the literature search service that you received (from submission of the search request to when you received the search results)?

Poor	Fair	Good	Very good	Excellent
O	O	O	O	O

27. Please provide suggestions for how your experience with our literature searching service could have been improved.
